# The Open Challenge of *in vitro* Modeling Complex and Multi-Microbial Communities in Three-Dimensional Niches

**DOI:** 10.3389/fbioe.2020.539319

**Published:** 2020-10-20

**Authors:** Martina Oriano, Laura Zorzetto, Giuseppe Guagliano, Federico Bertoglio, Sebastião van Uden, Livia Visai, Paola Petrini

**Affiliations:** ^1^Molecular Medicine Department (DMM), Center for Health Technologies (CHT), UdR INSTM, University of Pavia, Pavia, Italy; ^2^Department of Pathophysiology and Transplantation, University of Milan, Milan, Italy; ^3^Internal Medicine Department, Respiratory Unit and Adult Cystic Fibrosis Center, Fondazione IRCCS Ca’ Granda Ospedale Maggiore Policlinico, Milan, Italy; ^4^Department of Biomaterials, Max Planck Institute of Colloids and Interfaces, Potsdam, Germany; ^5^Department of Chemistry, Materials and Chemical Engineering “Giulio Natta” and UdR INSTM Politecnico di Milano, Milan, Italy; ^6^Technische Universität Braunschweig, Institute of Biochemistry, Biotechnology and Bioinformatic, Department of Biotechnology, Braunschweig, Germany; ^7^Department of Occupational Medicine, Toxicology and Environmental Risks, Istituti Clinici Scientifici (ICS) Maugeri, IRCCS, Pavia, Italy

**Keywords:** polymicrobial cultures, chronic infections, antimicrobial, antibiotic resistance, *in vitro* models, ecological models, lung dysbiosis, biofilm

## Abstract

The comprehension of the underlying mechanisms of the interactions within microbial communities represents a major challenge to be faced to control their outcome. Joint efforts of *in vitro*, *in vivo* and ecological models are crucial to controlling human health, including chronic infections. In a broader perspective, considering that polymicrobial communities are ubiquitous in nature, the understanding of these mechanisms is the groundwork to control and modulate bacterial response to any environmental condition. The reduction of the complex nature of communities of microorganisms to a single bacterial strain could not suffice to recapitulate the *in vivo* situation observed in mammals. Furthermore, some bacteria can adapt to various physiological or arduous environments embedding themselves in three-dimensional matrices, secluding from the external environment. Considering the increasing awareness that dynamic complex and dynamic population of microorganisms (microbiota), inhabiting different apparatuses, regulate different health states and protect against pathogen infections in a fragile and dynamic equilibrium, we underline the need to produce models to mimic the three-dimensional niches in which bacteria, and microorganisms in general, self-organize within a microbial consortium, strive and compete. This review mainly focuses, as a case study, to lung pathology-related dysbiosis and life-threatening diseases such as cystic fibrosis and bronchiectasis, where the co-presence of different bacteria and the altered 3D-environment, can be considered as worst-cases for chronic polymicrobial infections. We illustrate the state-of-art strategies used to study biofilms and bacterial niches in chronic infections, and multispecies ecological competition. Although far from the rendering of the 3D-environments and the polymicrobial nature of the infections, they represent the starting point to face their complexity. The increase of knowledge respect to the above aspects could positively affect the actual healthcare scenario. Indeed, infections are becoming a serious threat, due to the increasing bacterial resistance and the slow release of novel antibiotics on the market.

## The Need for Platforms for Polymicrobial Culture

Pure bacteria cultures are an unnaturally occurring scenario. Like all organisms, bacteria live as part of an ecosystem, sharing, exchanging and competing for resources with other microorganisms present in their environment (Freilich et al., 2010). This fact is combined with the mechanical, morphological and biochemical conditions of the microenvironment, leading to a biochemical action-reaction effect that changes the way bacteria communities respond to drastic events (Raimbault, 1998; Tuson et al., 2012). Specifically, bacterial ecosystems are mainly organized in three-dimensional matrices, either self-produced, like biofilms, either otherwise produced, like mucus. As a result, for example, *in vitro* pure bacteria culture in suspension in a medium (planktonic conditions) is significantly more susceptible to antibiotic treatment than an *in vivo* infection where the same bacterial strain is the dominant pathological agent. So much so that when a certain antibiotic is screened to be potentially effective to treat a patient, the needed antibiotic concentration to be delivered to the infection site must be 100 to 1000 times higher than the antibiotic concentration assessed in the susceptibility test (Macià et al., 2014).

These factual cues reveal the inability of *in vitro* culturing methods to provide the means for hosting the complexity of natural microbial environments. New technological and methodological tools are quested to enable the study of microbial communities in a reproducible and controlled manner, either *in vitro* or *in silico*. The aim of this is boosting the understanding of the dynamic mechanisms underlying the way that bacterial communities react and evolve in response to external stimuli and to study their delicate equilibrium within any environment, including the human body. These methods could work in synergy with proper *in vivo* models. Diverse applicative fields can benefit from the new methodological tools: the study of the effect of drugs, nutrients, prebiotics and probiotics on the gastrointestinal microbiota, or the effectiveness of antimicrobial treatments. The understanding of the underlying mechanisms precedes the deliberate control and modulation of the response, avoiding trial-and-error approaches based on phenomenological observations.

The development of new methods originates from the parallel study of the natural microbial environments that are still mainly unexplored. We will address the bacterial niches in the human body with a specific highlight on the lung environments and chronic and multiple infections, as they represent one of the most challenging issues to be addressed. In these cases, the changes in the microenvironment are related to the change of the dynamics of the multistrain interactions. This paper addresses the complexity of the polymicrobial systems in the human body, with a specific focus on challenging polymicrobial infections. We describe the state of the art of *in vitro* and mathematical models as powerful tools to investigate the natural complexity. Their multifaceted potential can influence diverse application fields. Among them, we focused on the fight against infectious diseases either by personalized testing of the efficacy of antimicrobial substances or the development of new therapeutic approaches.

## 3D-Bacterial Niches in the Human Body

Each species in an ecosystem is thought to occupy a separate, unique niche. The ecological niche of a microorganism describes how it responds to the distribution of resources and competing species, as well as how it alters those same factors in turn. The niche is a complex description of how a microbial species uses its environment. In nature, bacteria interact in complex communities, with the contemporary presence of different species whose abundance fluctuates over time in response to their mutual interaction and to the surrounding environment. Their interaction can lead to competition or cooperation to achieve an evolutionary advantage. The complex microbial communities, also known as microbiota, are fundamental in the human body development and the presence of dysregulation of the microbial community is associated with different disease states. Human gut microbiota, for example, has important functions in the development of immunity, defenses against pathogens, host nutrition including the production of short-chain fatty acids, synthesis of vitamins, making it an essential component of the human body (Bauer et al., 1963; Amon and Sanderson, 2017). Moreover, the colonization from specific microorganisms can protect from parasites or other pathogens: *S. epidermidis* on the skin, for example, promotes the production of transmembrane proteins and cytokines involved in the immune response and ultimately protects against infection with the parasite Leishmania major (Naik et al., 2012).

Various biological environments in the human body host microbial communities (Costello et al., 2012), yet, for its clinical relevance, our focus is on the lung environment and its pathologies.

### Lung Microbiota and Its Dysregulation in Pathological Conditions

Lungs were thought for many years as sterile, being the respiratory tract less charged in genetic material than other body districts, i.e., gastrointestinal or urinary tract. Nowadays, we know that the lungs have a physiological microbiome (Sze et al., 2015; Wang et al., 2016; Segal et al., 2017). Lungs are in constant communication with the external environment with continuous microbe immigration and elimination through mucociliary clearance, although a resident flora is present even in the lungs of healthy subjects (Hilty et al., 2010; Morris et al., 2013; Bassis et al., 2015; Dickson et al., 2015).

Lungs are not the ideal location for bacterial proliferation in comparison to the intestinal tract, because they are low-nutrient sites and their physiological conditions related to oxygen tension, pH, temperature and inflammatory cell infiltration can vary. These conditions determine a continuous change in the microbial pulmonary ecosystem (West, 1968; Ingenito et al., 1987).

The presence of lung microbiota was identified after the development of culture-independent techniques. The sequencing of highly conserved loci, like 16s rRNA gene, through high throughput sequencing, is the most common technique for microbiome identification. Moreover, the development of bacterial genomes databases allowed the scientific community to assign a relative abundance of bacteria and diversity (Costa et al., 2018).

Lung microbiota develops in early life and it is specific for the method of delivery. Vaginal-delivered children’s microbiota resulted to be mainly composed of *Lactobacillus*, *Prevotella*, or *Sneathia* species, while cesarean section born children acquired *Staphylococcus*, *Corynebacterium*, and *Propionibacterium* species (Dominguez-Bello et al., 2010). The early microbiota acquisition protects the lungs from responses to possibly inhaled antigens. Healthy lungs microbiota composition is dynamic and among the multiple bacteria present in the lungs, whose abundance and composition vary in time, bacteroidetes and firmicutes constitute a retained core (Erb-Downward et al., 2011; Morris et al., 2013; Segal et al., 2013).

Microbial diversity in the lungs seems to be crucial in the homeostasis of the respiratory system. Dysbiosis situations result in both acute and chronic diseases (Boyton et al., 2013; Surette, 2014; Mammen and Sethi, 2017).

**FIGURE 1:**
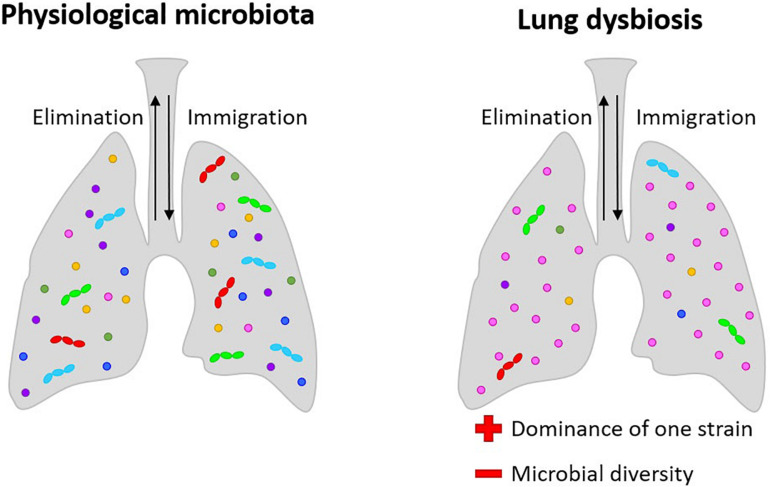
Lung microbiota: in physiological conditions, its composition is dynamic and among the multiple bacteria present in the lungs; in pathological conditions, microbial diversity is lost, and dysbiosis situations result in both acute and chronic diseases, with the dominance of a single or a small group of taxa. In bronchiectasis, dehydrated, thickened secretions lead to endobronchial infection with a limited spectrum of distinctive bacteria, mainly *Staphylococcus*, *Pseudomonas*, and *Burkholderia*. In CF, the dominance of *Pseudomonas aeruginosa* and *Burkholderia cepacea* are related to the progression of the pathologies.

The level of dysregulation may be correlated to disease severity and the dominance of a specific microorganism seems to be linked to the disease state (Venkataraman et al., 2015) (Figure 1). The predominance of a bacterial species in the lungs and a decrease of richness in microbiota seems to be associated with diseases like cystic fibrosis (CF) (de Dios Caballero et al., 2017; Zemanick et al., 2017). In both acute and chronic (bronchiectasis) diseases, the disease state is associated with the loss of bacterial diversity and the dominance of a single or a small group of taxa (Tunney et al., 2013).

Interestingly, chronic infections are correlated with a change in mucus viscosity, indicating the interdependence of the three-dimensional matrix and the bacterial interspecies interactions. In the typical bacterial niches of CF and bronchiectasis, the chronic co-presence of different pathogens is a life-threatening issue. CF is a systemic disease caused by genetic mutations in which pulmonary implications are strictly related to disease severity and mortality. It involves airway surface liquid depletion producing mucus obstruction and chronic inflammation with persistent leukocyte accumulation at the pulmonary level (Cheung et al., 2008). Dehydrated, thickened secretions lead to endobronchial infection with a limited spectrum of distinctive bacteria, mainly *Staphylococcus*, *Pseudomonas*, and *Burkholderia*, an exaggerated inflammatory response leading to the development of bronchiectasis, progressive obstructive airways disease and frequent exacerbations (Guggino and Banks-Schlegel, 2004; Marshall et al., 2016). *P. aeruginosa*, which is one of the most frequent bacteria in CF, is also able to generate biofilm. Biofilm formation leads to further difficulty in antibiotic treatment. *Burkholderia* instead could be very dangerous in CF, due to their intrinsic antibiotic resistance, increasing lung disease severity. Infection from *Burkholderia cepacia* complex is also an exclusion criterion for lung transplants. Besides, continuous exposure to antibiotics is contributing to the insurgence of multidrug-resistant pathogens and studies are supporting a loss of microbiome diversity with age, due to antibiotics use with a parallel increase of disease severity (Knippenberg et al., 2015).

Bronchiectasis is a chronic respiratory disease characterized by permanent airway dilation with daily symptoms such as cough, sputum production, and recurrent exacerbations. The prevalence of bronchiectasis is up to 500/100,000 individuals increasing worldwide (Amati et al., 2017).

Bronchiectasis leads to a decreased mucus excretion and an overgrowth of microorganisms and this condition causes inflammation in the lungs. Infection and inflammation, that easily become chronic, leads to further damage to the airways and worsening of patients’ conditions (Swenson et al., 2017). Bronchiectasis patients’ lungs are colonized by several pathogens like *Pseudomonas aeruginosa*, *Haemophilus influenzae*, *Moraxella catarrhalis, Escherichia species, Klebsiella species* (Gram-negative) and Gram-positive as *Streptococcus pneumoniae* and *Staphylococcus aureus* detected though standard microbiology (Chalmers et al., 2014). Bronchiectasis etiology is very heterogeneous and identified as post-infectious in 20% of patients, caused by chronic obstructive pulmonary disease (COPD) in 15%, 10% as a cause of tissue diseases, 5.8% due to immunodeficiency and 3.3% linked to asthma. Identified causes cover 60% of patients, while in 40% of cases the cause of bronchiectasis is idiopathic (Amati et al., 2017). Microbiome analysis of low airways samples shows pronounced domination of proteobacteria including *Pseudomonas* and *Haemophilus* in association with neutrophil-mediated inflammation. In opposition, in the presence of lower neutrophilic inflammation and frequent exacerbations, the microbiome was dominated by firmicutes (Rogers et al., 2014; Taylor et al., 2015; Chalmers et al., 2017). Genera detected in lower airways through 16s rRNA gene sequencing during a stable state are usually *Prevotella*, *Veillonella Streptococcus*, *Moraxella*, *Neisseria*, *Flavobacterium*, *Leptotrichia*, *Fusobacterium* (Rogers et al., 2014; Lee et al., 2018).

Being interspecies interactions and the microbial niches crucial for the physiological conservation of the body homeostasis, both basic and translational studies in this field can result in clinical advances in the treatment of many diseases.

## Antibiotic Resistance and Drug Development in Lung Diseases

The lung microbiota is an ecosystem that can enter a state of dysbiosis when a pathological bacterium becomes dominant. Now the use of antibiotics is the traditional medical approach to treat this condition. Antibiotics are very useful in medicine, but aside their effect on target cause effects on the entire bacterial community that take time to recover. The remaining bacterial community becomes in severe need for rehabilitation until a new microbiota eubiosis is achieved.

Besides that, the awareness around the development of bacterial resistance to antibiotics is growing. Antibiograms are the tool used by clinicians and scientists to evaluate the susceptibility of bacterial isolates to antibiotics and are fundamental in diseases like CF or bronchiectasis where patients are constantly facing chronic respiratory infections (Swenson et al., 2017). In reality, susceptibility indicates that the tested antibiotic can neutralize the pathological bacteria culture in planktonic conditions at a concentration that is far lower than what would be required for infections in humans, and, as a consequence, its ability to control the infections is overestimated. Whether or not the treatment is effective, non-pathological bacteria communities, crucial for maintaining the symbiotic functions of the human microbiome, are severely debilitated or acquire the ability to resist such antibiotic class (Zhang et al., 2019).

Moreover, some bacteria are also able to form biofilms. Biofilms are bacterial communities that can develop in nature as well as in the human body, well-coordinated communities in which bacteria live in a self-produced extracellular matrix composed of exopolysaccharides, DNA and proteins (Costerton et al., 1995). CF lungs are a favorable place for biofilm formation given the properties of CF mucus. Biofilms in CF lungs can increase the spreading of antibiotic resistance among the community and also limit the bioavailability of drugs by inhalation creating a physical barrier. As mentioned above, *P. aeruginosa* is one of the most dangerous and frequent bacteria in CF, able to install very resistant chronic infection and able to initiate biofilm formation (Moreau-Marquis et al., 2008).

### Development of Innovative Approaches for Combating Lung Infections

The use of antibiotics to fight bacterial infections seems not to be the only strategy to face this problem. An alternative strategy, which neutralizes the pathological bacteria while contemporaneously leading the lung microbiota toward eubiosis could revolutionize the way lung infections are treated. This is precisely what new approaches using pre- and probiotics are aiming at. Strategies that employ manipulation of distal (gut) and local (lung) microbiomes to aid the host in combating a lung infection are proposed (Dumas et al., 2018). One strategy concerns the use of the immune-modulatory ability of the gut (distal) microbiome to allow the host to gain back control over the lung infection. Previous studies demonstrated the feasibility to recreate lung dysbiosis in mice, followed by seeding pathological bacteria – *K. pneumoniae*, *S. pneumoniae* and *Mycobacterium tuberculosis* – to model a severe lung infection *in vivo*. After delivering a fecal transplant, they noticed that the host restored control over the lung infection through increased immune system activity (Khan et al., 2016; Schuijt et al., 2016; Brown et al., 2017). This is one of the many surprising systemic effects of the intestinal microbiota. Not long ago it was assumed that a healthy lung was a sterile environment, now there is evidence indicating that not only it exists but also microbiome from distal body districts (e.g., gut) influences the stability of lung microbiota (Dumas et al., 2018).

The immunomodulation effect of certain bacteria, e.g., *Lactobacillus genus*, toward the well-being of their host is not restrained to a distal body district (Barbieri et al., 2013, 2017, 2019). These studies focus on the potential of administering probiotics directly over the infected region, to understand if these can establish a presence within the microbiota under a state of dysbiosis and assist in restoring control over the infection. They have infected the lungs of mice with *S. pneumoniae*, followed by nasal administration of *Lactobacillus rhamnosus*. It was observed that *L. rhamnosus* stimulates and increases the number of B cells (Barbieri et al., 2013), CD4 + T lymphocytes (Barbieri et al., 2017), macrophages and dendritic cells (Barbieri et al., 2019) in the lung, significantly improving the outcomes of pneumococcal infections. Although results are promising, clinical trials are needed to help understand if probiotics can be used as an aid to antibiotics in humans. Nevertheless, it provides another potential pathway to help solve the antibiotic crisis that is currently unfolding due to the rise of antimicrobial resistance (Hendriksen et al., 2019), led by the inability of antibiotic development stakeholders to bring new antibiotic classes to the market.

The health industry deals with bacterial infections by developing new testing methods and tailored strategies to fight infections; developing new bacterial culturing tools could increase their efficacy and broaden their range of applications. Improvement of *in vitro* efficacy screening seems to have the potential to enhance the development of new treatments.

## *In vitro* Modeling the Three Dimensional and Multimicrobial Communities

**FIGURE 2:**
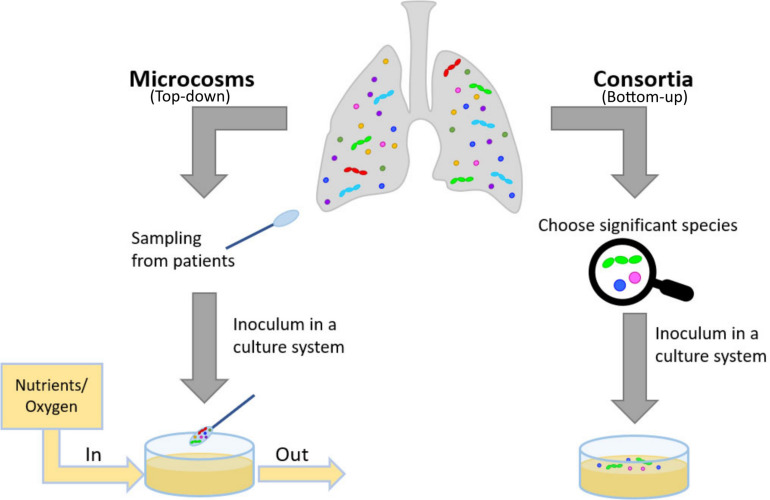
Description of how to model *in vitro* physiological and pathological situations. In a top-down approach, the patient’s pool of bacteria is collected and cultured, ideally preserving its multimicrobial nature. In a bottom-up approach, the complexity of the physiological or pathological situation is reduced by introducing the main features fundamental for the study, e.g., selecting significant species and culturing in an appropriate substrate, possibly reproducing the 3D-environment of the mucus.

*In situ* models would be the ideal solution to study biota and biofilm behavior in their native environment (Wimpenny, 1997) and test for novel antibiotics. Yet, in the case of bacterial related infection, such models are hardly attainable. Thus, researchers usually turn to *in vitro* and *in vivo* models. Focusing on the first type, there are two main approaches: a top-down approach, microcosms and a bottom-up approach, consortia (Figure 2) (Sissons, 1997; Wimpenny, 1997). In the microcosm approach, biological materials mimicking the target environment evolve *in vitro*: polymicrobial cultures from the natural biota, properly sampled from voluntary donors, are often used (Sissons, 1997; Lebeaux et al., 2013). In theory, this approach allows obtaining the most closely related model to the native conditions, but it is highly affected by inter- and intra-variability of donors and it can be easily influenced by the sampling methods (Sissons, 1997; Lebeaux et al., 2013). Moreover, among the sampled bacteria, strict anaerobes do not easily survive standard culture techniques (Woods et al., 2012) and the resulting flora of the model could be less well-representative of the native environment than initially thought. To study specific aspects of bacterial interactions, to exploit even simpler experimental models selecting the most relevant species to target a scientific question: this bottom-up approach can be labeled as ‘consortium’ because among the whole fauna, only a few species will be used. Well-established examples of bacterial consortia are the six and nine species ‘Marsh’ consortia to model dental plaque (Sissons, 1997; Brown et al., 2019). Both microcosms and consortia can be classified as open (continuous renovation of nutrients, removal of metabolic by-products and aerial exchanges allowed) or closed (bacteria grow on a limited supply of nutrients) (Lebeaux et al., 2013). Open models are sometimes referred to as ‘dynamic models’: this definition is widespread in microbiological studies [e.g., in studies using CDC biofilm reactor (Bilal et al., 2019)]. Yet, despite having a continuous medium flow, open bioreactors operate in a stationary state. Therefore, here we will refer to such systems as ‘open’ and will use the term ‘dynamic’ for the transient and unstable interactions between microorganisms that will ultimately lead to a new steady-state, different from the initial conditions of the system. The culture conditions deeply affect the response of bacteria to external stimuli, including the effect of antibiotics. The challenge is to produce realistic tools for the study of bacterial interactions and drug discovery. These tools should provide not the complete physiological situation, but the key features relevant for each aim. There is not “the ideal” *in vitro* model of lung microbiota, as it is strictly dependent on the feature to be tested. In antibiotic resistance studies, e.g., the 3D-matrix is relevant for permeability studies (Pacheco et al., 2019) but also to allow the formation of self-protecting clusters of bacteria (Melaugh et al., 2016). We propose a bottom-up approach in recapitulating the physiological complexity of the microbial niches within *in vitro* models. This approach implies to deconstruct in pieces the complex physiological situation by employing different tools, such as 3D models, the *in vitro* cultures of more than one bacterial species. Mathematical and ecological models complete the picture, by rendering the competing effects of the co-presence of different species.

### Modeling the 3D-Microenvironment: *in vitro* Studies of Biofilm-Embedded Bacteria

Biofilm is the most common form of life of bacteria on Earth: it represents one of the most non-treatable and recalcitrant forms of infections when considering human health. As an example, *P. aeruginosa*, that is associated with clinical decline and severe outcomes both in cystic fibrosis and in bronchiectasis, is a biofilm-forming bacterium. The same strain is often involved in chronic wounds, together with *Staphylococcus aureus* and *Clostridium perfringens* (Woods et al., 2012; Bertesteanu et al., 2014). Experiences in the literature show resistance of these bacteria to eradication. The inefficacy of the treatment is often believed to be due to established antibiotic-resistant biofilms (Moreau-Marquis et al., 2008). The study of infections, in some cases, reflects a limited perspective, mainly analyzing the behavior of a few well-characterized, pure laboratory bacterial strains. The strategies employed to reproduce *in vitro* biofilms are examples of 3D-models for bacterial cultures. Pharmacodynamic parameters, as well as culturing devices and analytical methods, will be presented and discussed.

The main classical parameters (Macià et al., 2014), specifically used for planktonic bacteria, are still adopted to analyze the effect of different compounds on biofilm, namely the Minimal Inhibitory Concentration (MIC) and the Minimal Bactericidal Concentration (MBC) (Table 1). Minimal Biofilm Inhibitory Concentration (MBIC) and Biofilm Bactericidal Concentration (BBC) are indeed the two parameters that parallel MIC and MBC but specifically accounting biofilm. Two further parameters have been harnessed to analyze biofilm responses to external compounds: Biofilm Preventing Concentration (BPC) and Minimal Biofilm Eradication Concentration (MBEC) (Table 1). BPC refers to the preventive effect of an antibiotic in biofilm formation. Therefore, bacteria are seeded together with the compound under analysis and biofilm growth is monitored (Fernández-Olmos et al., 2012). Nevertheless, MBIC and BPC are reported as the lowest concentration of drug that resulted in an OD_650_ nm difference of ≤ 10% of the mean of two positive control well readings. Contrarily to BPC, the three parameters MBIC, BBC and MBEC are analyzed on biofilms already formed, subsequently challenged with the drug. MBEC represents the lowest concentration of an antibiotic that prevents visible growth in the recovery medium used to collect biofilm cells. These parameters are useful to compare results among different laboratories, but international guidelines on biofilm are missing, unlike for planktonic cultures (Clinical & Laboratory Standards Institutes [CLSI], 2019). Examples of reproducibility across different laboratories are available (Parker et al., 2014) but a general lack of standards in anti-biofilm compounds testing is evident.

**TABLE 1:** Pharmacodynamic parameters employed to analyze the effect of antimicrobial substances, on both planktonic tests and tests accounting biofilm.

*Effect*	*Acronymous*	*Definition*	*Mode of growth*
Inhibitory effect	MIC	The lowest concentration of an antibiotic that inhibits the visible growth of a planktonic culture after overnight incubation	*Planktonic*
	MBIC	The lowest concentration of an antimicrobial substance at which there is no time-dependent increase in the mean number of biofilm viable cells when an early exposure time is compared with later exposure time (OD_650_ nm difference of ≤ 10% of the mean of two positive control well readings)	*Biofilm*
	BPC	The lowest concentration of an antimicrobial substance at which there is no time-dependent increase in the mean number of biofilm viable cells when bacterial inoculation and antibiotic exposure occur simultaneously (OD_650_ nm difference of ≤ 10% of the mean of two positive control well readings)	*Biofilm*
Bactericidal effect	MBC	The lowest concentration of an antibiotic able to produce a 99.9% CFUs reduction of the initial inoculum of a planktonic culture	*Planktonic*
	MBEC	The lowest concentration of antimicrobial agent that prevents visible growth in the recovery medium used to collect biofilm cells	*Biofilm*
	BBC	The lowest concentration of antimicrobial agent that killed 99.9% of the cells recovered from a biofilm culture compared to growth control	*Biofilm*

*The Table was reformulated from reference 4 (Copyright Sep 11, 2020) with permission from Elsevier (licenses 4905821434139). CFU (Colony Forming Units).*

Aside from the different parameters that can be tested, a panel of experimental setups can be deployed to investigate biofilm-embedded bacteria (Table 2). These culturing devices are in general divided into closed and open systems, the former being mostly related to microtiter plates, the latter involving dynamic systems underflow. Closed systems are in the vast majority of cases, static, batch cultures that are analyzed either during the experiment or at the endpoint. These systems allow scale-up of the analysis, involving more compounds at different concentrations at a single time, thanks to the use of microtiter plates. Furthermore, these systems are inexpensive and allow quick readout of the test. Nevertheless, the main limitations of these approaches involve great variations between wells (therefore, usually, at least four replicates for each condition are tested), incomplete disruption of biofilm upon endpoint analysis and presence of exhausted nutrients in the well until the end of the test.

**TABLE 2:** Schematic overview of the strategies available to study biofilm communities.

Type	Main Features	Advantages	Drawbacks	Example systems	Evaluated aspects	Study
Closed	Static cultures. Realized within batches and microtiter plates	Low price. Possible scale-up of the analysis High throughput	Low adherence to *in vivo* situations High variance Exhaust medium hard to be eliminated. Incomplete disruption of the biofilm	Calgary Biofilm Device	Growth and shedding of biofilm on a bump, immersed in a bacterial suspension	Harrison et al., 2010; Chavant et al., 2007
				BioFilm Ring Test^TM^	Early biofilm formation is quantified analyzing the precipitation kinetics of a paramagnetic bead through the biofilm itself	Peterson et al., 2015
Open	Dynamic cultures. Strong reliance on bioreactors and pump systems	Realistic experimental conditions Continuous refresh of the medium	High complexity Low throughput Expensiveness	Flow Chambers	Bacteria behavior in presence of physiological-like stimuli	Gómez-Suárez et al., 2001; Pamp et al., 2009; Jass et al., 1995
				Modified Robbins Device	Biofilm produced on a given specimen by a log-phase broth culture	Goeres et al., 2009
				Drip Flow Biofilm Reactor	Biofilm produced on a given specimen under low fluid shear	Donlan et al., 2004
				BioFlux	Long term bacteria-bacteria and bacteria-environment interactions	Lopes et al., 2018

Three main supports have been harnessed to study biofilm-forming bacteria in closed systems: apart from plain 96-well tissue culture plates (O’Toole and Kolter, 1998; Stoodley et al., 2002; Ren et al., 2014; Pallavicini et al., 2017), Calgary Biofilm Device (CBD) and BioFilm Ring Test^TM^ belong to this category. CBD is composed of a microtiter reservoir plate that perfectly matches a lid with pegs that can be accommodated in the reservoir chamber (Ceri et al., 1999; Harrison et al., 2010). Biofilm grows on the suspended pegs. This system allows contemporary screening of many conditions, reduced volumes and contaminations since little handling is required upon lid transfer. The third set up, BioFilm Ring Test^TM^, has been instead designed to investigate early biofilm stage formation (Chavant et al., 2007). Paramagnetic beads are co-inoculated upon biofilm seeding and depending on their precipitation or not after an initial incubation, they indicate if the biofilm has formed or not. If the community is starting to build its extracellular matrix, beads are trapped and cannot be therefore attracted toward the bottom of the microtiter plate by a magnet. Instead, if a visible spot appears at the bottom of the well, this indicates that beads can precipitate and therefore the biofilm formation has been hampered.

Concerning open systems, all the devices include a pump, which allows flux of nutrients, and bacterial cells, thus mimicking more realistic conditions, both for environmental and for healthcare-related biofilms. Shear forces are indeed one of the parameters that can induce stress and modify bacterial behavior (Stoodley et al., 2002; Purevdorj et al., 2002; Peterson et al., 2015). Furthermore, fresh medium is provided, while eliminating exhausted one, thus again increasing mimesis of natural environments. Compared to closed systems, open ones guarantee a more complex setup, having as a drawback a reduction in throughput, since not many conditions can be analyzed at the same time. In general, these systems require more expensive settings and maintenance.

Up to now, flow chambers are among the most convenient tools to understand how the embedded bacteria behave in the presence of flux because direct visualization of the samples is possible since the transparent devices can be coupled to microscopes (either optical, fluorescence or confocal) (Gómez-Suárez et al., 2001; Beyenal et al., 2004; Pamp et al., 2009). Other flow cells that allow parallel testing of different conditions exist, although, in this case, direct imaging is not allowed. These systems are the Modified Robbins Device (Jass et al., 1995) and the Drip Flow Biofilm Reactor (Goeres et al., 2009). Conditions during the experiment can be varied and samples collected thanks to the presence of different valves. Different surfaces can be accommodated within the devices, thus allowing investigation of anti-biofilm surfaces. Imaging is allowed only as an endpoint experimental measure.

The Center for Disease Control Biofilm reactor was named after their inventors (Donlan et al., 2004) and allows testing of up to 24 different coupons in high shear since the system is rotating. Coupons can be easily removed and thus analyzed at different intervals.

Finally, more recently, microfluidic devices have been developed. They can be, to some extent, designed and tailored to each experimental need. These systems are physiologically closer to the conditions that microbial communities may encounter, thus providing a more reliable experimental set up (Drescher et al., 2013; Rusconi et al., 2014). Besides reductions in equipment and reagents, microfluidics allow in-line analysis. Recent advances allowed designing a 96-well plate with connecting microfluidics channels, named BioFlux (Benoit et al., 2010), for throughput analysis.

Different techniques can be employed to analyze biofilm-embedded bacteria (Table 3). Viability is one of the first aspects that is analyzed in biofilms. To get quantitative data, the method that implies the count of viable bacteria able to form colonies when re-plated on agar (colony forming units, CFU) is still the most adopted yet laborious method. Especially in the multispecies community, recognition of single species ratios is challenging because it requires specific differential and selective agar media, which are not always available and may be expensive (Røder et al., 2016; Lopes et al., 2018). Other quantitative methods rely on metabolic assays in which viable bacteria can transform substrates that may change or turn into a colored product: examples are tetrazolium salts (e.g., XTT or MTT) or resazurin based products (Van den Driessche et al., 2014; Alonso et al., 2017). Other reagents are used to stain biofilms. Crystal violet unspecifically colors extracellular polymeric substance and cell, thus providing broad information on biofilm thickness and growth (Sharma et al., 2008). If instead general information of viable cells is required, staining with LIVE/DEAD BacLightTM Bacterial Viability Kit (Invitrogen) can be performed and visualization under a Confocal Laser Scanning Microscope (CLSM) would also provide the spatial distribution of bacteria. SEM (Golding et al., 2016; Gomes and Mergulhão, 2017; Huang et al., 2020) can provide useful information with a proper sample preparation. Innovative methods employ cryo-SEM and Environmental SEM to avoid dehydration. Approaches that may allow to specifically tag different components of the matrix, while advanced chip microfluidics are leading toward the development of methods with higher throughput (Di Poto et al., 2009; Hansen et al., 2019; Hartmann et al., 2019). Further methods can be applied to differentiate among species, e.g. probing the biofilm with specific Fluorescence *In Situ* Hybridization (FISH) or with species-specific primers with quantitative qPCR (Amann and Fuchs, 2008; Almeida et al., 2011; Azevedo et al., 2011; Ren et al., 2014; Lopes et al., 2018). These approaches are destructive and are carried out as endpoint analysis. An overview of the most used techniques to study biofilm embedded bacteria is reported in Table 3.

**TABLE 3:** Schematic overview of the strategies available to study biofilm embedded bacteria.

Assay	Type	Principle	Features	Advantages	Drawbacks	Study
CFU count	Viability	Diluted bacteria are plated on agar media and incubated until colonies growth; colonies formed are counted	Direct quantitative evaluation of viable cells	Quick and not expensive	Difficult to evaluate multispecies community Specific differential/selective media are needed to evaluate different bacteria The CFU countable range is relatively narrow, errors may arise from biofilms (high cell aggregation)	Røder et al., 2016; Alonso et al., 2017
XTT, MTT	Metabolic assays	Cells are incubated with a substrate that is metabolized by cells in a colorigenic compound	Indirect quantitative evaluation of viable cells	Mostly used with in planktonic situation, quantitative, relatively expensive	The use of specific compounds may interact with the substrate Biofilm embedded bacteria may have a different metabolic activity than planktonic bacteria usually used as standard	Van den Driessche et al., 2014; Sharma et al., 2008
Crystal violet	Biofilm staining	The compound stains the biofilm making it visible	Direct measure of biofilm mass	Provides broad information on biofilm thickness and growth	The entire biofilm mass is stained (extracellular matrix and cells), no information on cell viability is given	Golding et al., 2016
Live/dead staining	Biofilm staining	The compound differentially stains cells based on the integrity of the membrane	Direct quantitative measure of viable cells	Mostly used in planktonic situation, may provide the spatial distribution of bacteria in a biofilm	High-throughput quantification of biofilm viability may be difficult The use of CLSM is needed	Gomes and Mergulhão, 2017
FISH	Species differentiation	Species specific fluorescent probes hybridize with bacterial oligonucleotides making bacteria visible	Direct qualitative visualization of different strains	The use of a different probes specifically distinguishes among bacteria	If the probe target is low, the signal may be not detectable against the background	Parker et al., 2014; Røder et al., 2016; Hansen et al., 2019; Hartmann et al., 2019; Almeida et al., 2011
qPCR	Species differentiation	PCR amplification of a target release fluorescence proportional to the initial bacterial load	Direct quantitative or semi-quantitative visualization of bacteria	The use of a different probes specifically distinguishes among bacteria	It does not distinguish among viable and non-viable cells	Ceri et al., 1999; Røder et al., 2016
Microbiome	Next generation sequencing	Next-generation sequencing target amplification of 16s rRNA gene	Direct relative analysis of microbial community	Untargeted and relatively expensive, high-throughput	Not quantitative, bacteria identification to genus	Sorek and Cossart, 2010
Shotgun metagenomics	Next generation sequencing	Next-generation sequencing of genes in all bacteria	Direct relative analysis of microbial community and bacterial features	Untargeted, acquisition of all the genetic information in the bacteria	Very expensive High bioinformatic expertise needed for data-analysis	Amann and Fuchs, 2008; Sorek and Cossart, 2010
Metatran scriptomics	Next generation sequencing	Next-generation sequencing approach to study gene expression of profile of the whole bacterial community	Direct relative analysis of bacterial gene expression	Untargeted, acquisition of all the gene expression of sequenced bacterial community	Very expensive High bioinformatic expertise needed for data-analysis	Azevedo et al., 2011; Liu et al., 2019; Sorek and Cossart, 2010
SEM	Morphological observation	Scanning electron microscopy observations. Samples fixed and gold sputtered. Innovative preparative steps of the sample and alternatives to gold sputtering. Possible wet-SEM and cryo-SEM to avoid dehydration steps.	Morphological and spatial analysis of both the three-dimensional matrix and the embedded bacteria	High resolution and magnification. High depth of field Suitable for analyses on heterogeneous surfaces Possibility to identify the type (in some case the genus) of the microbe	No viability informations The use of fluorochromes is not allowed The output of the analysis is strictly dependent on not-obvious preparative steps Low sensitivity unless concentrated samples used	Golding et al., 2016; Gomes and Mergulhão, 2017; Huang et al., 2020

*CFU, colony forming units; CLSM, Confocal Laser Scanning Microscope; FISH, fluorescence *in situ* hybridization; qPCR, Real time PCR; SEM, Scanning Electron Microscopy.*

Among high-throughput techniques recently developed we can find the so-called –omics technologies. Some of these techniques are genomics, metagenomics, microbiomic, transcriptomics and proteomics, metabolomics (Yates et al., 2009; Sorek and Cossart, 2010; Moree et al., 2012; Krohn-Molt et al., 2013; Liu et al., 2019). These assays can give access to an extensive amount of information, considering bacteria and bacterial genomes, as well as the entire set of transcripts, proteins and metabolites in a target niche (Yates et al., 2009; Sorek and Cossart, 2010; Moree et al., 2012; Krohn-Molt et al., 2013; Liu et al., 2019). Schematic information on some of the most used -omics techniques to study bacterial communities are reported in Table 3. A multi-omics approach is very used in translational research in the study of the interaction between bacterial niches and host response in human diseases (Hasin et al., 2017).

### Modeling the Interactions of Different Bacterial Strains: Co-cultures

Bacteria are usually considered as single-cell organisms and their ability to interact and form monospecies biofilms is quite often overlooked. The use of *in vitro* co-culture, a family of laboratory techniques that aim at growing two or more different cell types on the same support, is the current strategy to study multistrain interactions. The general purpose of co-culture is to recapitulate *in vitro* key communication and interaction mechanisms, which might intervene *in vivo* among the target cell types (Bogdanowicz and Lu, 2013), thus enabling the uncovering of crucial phenomena to design new therapies. Three macro-groups of co-cultures are distinguished based on the kind of cultured cells. Firstly, co-cultures of eukaryotic cells are realized by growing different cell types to either study physiological interactive processes (Bam et al., 2015; Jia et al., 2017; Yin et al., 2017) or develop a functional construct to be used in tissue engineering approaches (Vuornos et al., 2019). Similarly, co-cultures of prokaryotic cells have also been realized, in which various bacterial strains are cultured for many objectives, spacing from the study of interspecies *quorum-sensing* occurrences (An et al., 2006) to the industrialized production of chemicals (Jones and Wang, 2018). Hybrid co-cultures have also been proposed, in which both eukaryotic and prokaryotic cells are cultured, in the attempt to understand physiological symbiosis relations (Haller et al., 2000), or pathological effects on tissues after the settlement of an infectious phenomenon (Kim et al., 2010).

Co-cultures of prokaryotic cells are crucial for the development of new therapies against threatening diseases that are characterized by the presence of complex infectious microbiomes (e.g., cystic fibrosis, bronchiectasis, tuberculosis). However, the realization of fully functional and effective models is still challenging due to both the complexity of the *in vivo* microbiome itself, and the compelling boundary conditions stated by the host organism. Moreover, due to the novelty of the topic, a consensus on one platform for the realization of bacterial co-cultures does not exist yet. As a result, in our opinion, different studies relate to different protocols to be developed in a time-consuming quest of the appropriate experimental conditions. Moreover, in consequence, the results are difficult to compare and the whole picture to be reconstructed is rendered in parts that, in some cases, are difficult to match. Up to now, it is not possible to culture the whole microbiota and this challenge is far to be met.

## Mathematical and Ecological Models for Microbial Co-Cultures

**FIGURE 3:**
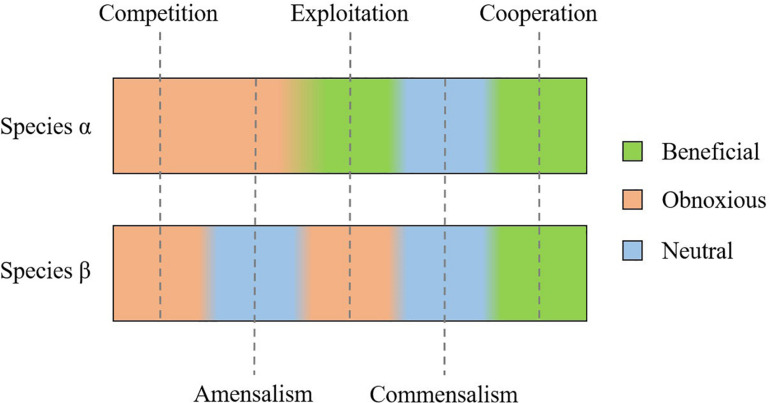
Possible interaction between two bacterial species (α and β): competition and cooperation are respectively obnoxious or beneficial for both species. Exploitation is beneficial for species α, at the expense of species β; whereas amensalism and commensalism do not affect species β, but are respectively obnoxious or beneficial to species α.

Mathematical models have characterized single-strain bacterial genetic evolution (Hindré et al., 2012) and antibiotic resistance (Greulich et al., 2015). Once validated, such models allow us to explore many different scenarios in a cost-effective way (Hindré et al., 2012; Allen and Waclaw, 2016). This possibility becomes of interest when modeling polymicrobial consortia facing the fact that bacteria do not live in monocultures in nature. In mixed communities, the most common natural ecosystems, each and every species optimized for different functions (Kneitel and Chase, 2004), and the interaction between different species sharing metabolic resources, defined as cross-feeding (Estrela et al., 2012; Coyte et al., 2015), has been modeled. Ecological theories in the framework of microbial consortia engineering aimed at designing efficient co-cultures for biofuel production or enhanced mineral recovery (Bernstein and Carlson, 2012). These theories have been applied also to human microbiota and polymicrobial infections (Costello et al., 2012; de Vos et al., 2017). In particular, it is possible to distinguish five different ecological interactions, when cross-feeding is involved (Figure 3; Coyte et al., 2015; Bernstein and Carlson, 2012; Faust and Raes, 2012):

– competition for space and resources;
– amensalism: one organism is inhibited or destroyed while the other organism remains unaffected;
– exploitation: one organism takes advantage of the other for resources scavenging and metabolism;
– commensalism: neither of the bacteria benefits from the other or provokes any harm;
– and cooperation: both the organisms benefit from the relation.

Mathematical models aim to relate the physical characteristics of the studied system and its dynamic evolution starting from the initial conditions until the system reaches an equilibrium. In case there are two bacterial strains and they coexist at the equilibrium, a bistability condition is reached (El Hajji et al., 2009; Wang and Wu, 2011; Assaneo et al., 2013). Considering the basic example of a chemostat, it is possible to model it with a system of five equations that describe the interaction between the bacteria and their nutrient support and consumption (Speirs et al., 1996). The outputs of the model are the variation in time of the bacterial population density and the nutrient concentration. The system of equations relates such variation with the system working conditions (e.g., initial population density of the bacterial species involved, growth rate, nutrient flow and intake) (Speirs et al., 1996). In this case, however, the relation between stability condition and model parameters is not straightforward, for the high the number of parameters required to solve the system of equations. To simplify it, it is possible to use the Lodka-Volterra equations that were initially designed to describe just the prey-predator competitive interaction (Lotka, 1922; Volterra, 1928), but they were generalized to all kinds of ecological interactions (May, 1973; Solé and Bascompte, 2012). This model consists of fewer equations than the physical model and a limited number of parameters that retain a physical interpretation of the model characteristics (Vet et al., 2018). Indeed, the Lodka-Volterra model will consist of as many equations as many are the species involved in the chemostat (e.g., two competing species will result in a model with two equations). Thanks to this simplification, it is easier to explore the relation between the initial conditions and the possible multiple species coexistence at the equilibrium (Vet et al., 2018). The model relies on some simplifying hypothesis: it ignores the mechanisms of interaction (like predator preferences) and it is based on the additivity assumption (Momeni et al., 2017). The latter assumes that pairwise interactions are sufficient to describe microbial community dynamics: the fitness of the polymicrobial system results from the sum of the fitness of each individual species, given by the interaction of the aforementioned species and the other interacting species (Momeni et al., 2017). Since it has been recently proven that the additivity assumption may fail, due to the complex non-linear interactions present in microbial communities (Dormann and Roxburgh, 2005; Momeni et al., 2017), it is possible to use higher-order models (Bairey et al., 2016) or models closer to the physical systems they want to describe (Vet et al., 2018), but at the expense of model simplicity.

Considering a relevant case of polymicrobial infections as Cystic Fibrosis, clinical data has been gathered regarding the microbial populations present under different conditions (age, use of antibiotics) (Klepac-Ceraj et al., 2010; Sherrard et al., 2019). In the near future, these databases could be exploited to validate mathematical models able to predict the development of infections in CF patients, knowing the patient history and, therefore, to choose the best treatment for each clinical case.

## Focus on *in vitro* Models for Cystic Fibrosis

Cystic fibrosis (CF) is one notable example of how the absence of well-established laboratory protocols for the realization of bacterial co-cultures drives researchers to choose and optimize different methods and culture conditions that are case-dependent systems, which challenges the comparison of data among different laboratories. One of the most pernicious aspects of this disease resides in the high number of species composing the infectious microbiome (Huang and LiPuma, 2016), resulting in a dynamic evolution of the chronic pulmonary infections throughout the life of the patients. Such behavior is one of the main factors causing the inefficiency of a classical antibiotic therapy in completely eradicating lungs-resident pathogens. Various *in vivo* models have been developed to test antibiotics efficacy: an essential requirement for such a model is the progression from a spontaneous bacterial infection to a chronic stage associated with biofilm formation (Lebeaux et al., 2013). *In vitro* models, on the other hand, can be useful to elucidate different aspects, such as the competitive effects of the different species on their viability and the modification of genic expression due to gain resistance to antibiotic treatments. The factors promoting the coexistence of different pathogens, such as phenotype modification, and their adaptive evolution to accommodate different species are also complex aspects that need to be understood and controlled. Most of the studies are conducted by dual cultures, with *S. aureus* and *P. aeruginosa*.

It is well known that *S. aureus* is the main colonizer of lung mucosa from the childhood of the cystic fibrosis patients, until the establishment of *P. aeruginosa* colonies, which tend to prevail in *S. aureus.* Despite the majority of *in vitro* models assuming *P. aeruginosa* to carry on complete eradication of *S. aureus*, it is uncommon to isolate both bacterial species from Cystic Fibrosis-infected lungs. In an *in vitro* model, it has been demonstrated that the culture in dual-species biofilm led to a consistent decrease of *S. aureus* relative abundance without achieving its complete eradication (Woods et al., 2018). This study exploits both a relatively simple closed multiwell model and a microfluidic system based on the culture of the strains on silicon tubes with a continuous circulation of fresh medium (Woods et al., 2018). The same model was previously used by the same authors to study the effect of signaling molecules on the genetic expression of *P. aeruginosa* monocultures (Davies and Marques, 2009; Marques et al., 2014). In the open model, bacteria grow at the liquid/solid interface: this configuration is gradually adding complexity concerning the basic multiwell closed model, but it is still far from the lung environment.

Two different co-culture systems were able to provide a deeper and more specific model for the early interaction of the two strains. It was supposed that *P. aeruginosa* genes, responsible to produce substances inducing the elimination of *S. aureus*, were downregulated when grown in dual-species biofilm. Both single and dual-species biofilms of *S. aureus* and *P. aeruginosa* were grown. To test this hypothesis, two experimental conditions were considered. In the first one *P. aeruginosa* was introduced at a specific proportion, that could mimic the *in vivo* situation in which a small amount of *P. aeruginosa* encounters a bigger and well-established amount of *S. aureus*, after the development of *S. aureus* biofilm. In the second, bacterial biofilms were co-cultured since the beginning of the experiment. While the previous example investigated the responses produced by the co-culture of bacteria in the mid-term (days), another research studied the reactions triggered at a genetic level during a short period (hours) of co-culture. In particular, various genomic analyses revealed how the competition for resources drives the first responses. The encounter of the two pathogens stimulated the up-regulation of genes related to the optimization of all those factors functional to the adaptation of metabolism, to excel in the competition for resources (Tognon et al., 2019). Pure cultures of *P. aeruginosa* and *S. aureus* were thus produced, and co-cultures were prepared by mixing the previously formed monocultures. Genomic analysis to evaluate changes in genic expressions of both organisms subsequently performed via RNA extraction, RNA-seq assays, and quantitative. Real Time-PCR (qPCR) (Tognon et al., 2019). In this case, a closed multiwell system was implemented, and single and co-cultures were seeded on solid agar plates enriched with nutrients and ions to sustain bacterial growth and mimic physiological conditions. The medium was tailored in previous studies to ensure a similar growth rate of both species (Tognon et al., 2017). Despite being elementary, the growth of the biofilms at the solid/air interface reproduces the pulmonary environment more than a microfluidic system.

The realization of *in vitro* co-cultures recently also played a key role in studying the causes of several evolutive changes observed in *P. aeruginosa* adaptation to the CF lung environment, like downregulation of lipopolysaccharide (LPS) and quorum sensing (QS) factors. *P. aeruginosa* might undergo two highly represented mutations (Tognon et al., 2017). The first one (WSP-level mutation, negatively correlated with *S. aureus* survival rates) was supposed to be caused by the culture condition (growth in a low oxygen environment) since it was present in both mono-cultured and co-cultured bacteria. The second mutation (a mutation at LPS level, increasing *P. aeruginosa* antibiotic resistance) appeared instead only in co-cultured *P. aeruginosa*, thus it has been thought to be an adaptive response to the presence of *S. aureus*. Such an experiment has been performed through the establishment of a peculiar protocol, in which *P. aeruginosa* has been evolved for at least 150 generations in liquid close multiwell cultures, both in the presence and absence of *S. aureus*, since interactions between *P. aeruginosa* and *S. aureus* are thought to be crucial in this process of adaptation. Serial dilutions of cellular suspensions were performed to mimic the *in vivo* situation in which a small amount of *P. aeruginosa* encounters pre-established large colonies of *S. aureus.*

Moreover, an interesting mutation of *P. aeruginosa* might take place in CF patients’ lungs, characterized by a switch from the native phenotype toward the so-called “mucoid” phenotype, which owes its name to the overproduction of alginate (Pritt et al., 2007). *P. aeruginosa* was demonstrated to undergo this phenotypic shift, expressing the mucoid phenotype not only characterized by the up-regulation of genes responsible for alginate production, but also by the down-regulation of genes encoding for virulence factors responsible of killing *S. aureus* (Limoli et al., 2017). Their protocol relied on the “agar-plate cross streak assay” (Balouiri et al., 2016). More in detail, *P. aeruginosa* isolated from mono-infected and co-infected patients was cross-streaked to a laboratory strain of *S. aureus.* In the same way, *S. aureus* clinical isolation was cross-streaked to a laboratory strain of *P. aeruginosa* (Limoli et al., 2017). This model, therefore, is in between the consortium and microcosm classification, as it sources parts of the bacteria from patients and partially from laboratory culture.

Another field taking advantage of *in vitro* co-cultures is the research against antibiotics resistance. Clinical data supported the hypothesis that the exposure of *S. aureus* to *P. aeruginosa* by-product leads to a drastic decrease in vancomycin (frontline antibiotic to treat *S. aureus* infections) efficiency against *S. aureus* (Filkins et al., 2015). This phenomenon was examined and it was found out that in the presence of the *P. aeruginosa* supernatant, vancomycin activity was reduced, and more colonies were detected whether *S. aureus* was cultured in planktonic or as biofilms. Given that *P. aeruginosa* tends to over-compete *S. aureus* while co-cultured (both *in vivo* and *in vitro* models), co-cultures were prepared in a peculiar way called “biofilm disruption assay” (Orazi and O’Toole, 2017). This procedure relied on the preparation of *S. aureus* monocultures. Then the biofilm produced by *S. aureus* pure cultures was mechanically disrupted, and *P. aeruginosa* supernatant was joined. This method compared to the Minimal Bactericidal Concentration (MBC) assay for planktonic cultures and as an alternative to the other methods to assess antibiotic resistance in biofilms.

Eventually, considering the role played by biofilm formation, the ability of *P. aeruginosa* to form biofilm was studied. This ability was investigated through scanning electron microscopy and confocal microscopy of different *P. aeruginosa* strains on an airway epithelial cell (Woodworth et al., 2008). Biofilm-forming strains were able to produce viable biofilms on the surface of airway epithelial cell monolayer. Further, *P. aeruginosa* and *V. Cholerae* were cultured on a monolayer of endothelial cells to assess the mechanobiological effect on the cells (Cont et al., 2020). In these cases, the co-culture is represented by the simultaneous culture of prokaryotic and eukaryotic cells. Despite being far from the physiological environment, such systems can be considered a microcosm for the complexity introduced by this type of co-culture.

It is glaring how the realization of a reliable *in vitro* model represents nowadays a crucial point for better understanding, and, consequently, try to solve as many criticalities as possible of such a polyhedral disease. However, comparing the experiments considered, it is possible to notice a remarkable heterogeneity under various points of view (e.g., duration of cultures, culture medium, and seeding ratios) (Table 4). Moreover, most of the experiments refer to planktonic cultures, without considering the case of bacteria grown inside some three-dimensional matrix mimicking the pulmonary CF mucus, which is known to be crucial in influencing bacterial behaviors. Regrettably, the realization of both a standard procedure and support, allowing the realization of co-cultures, still represents a big challenge. This reason is pushing scientists to realize culture systems tailored for the experimental purpose.

**TABLE 4:** Main features of recent experiments designed to explore bacterium–bacterium interactions in the case of cystic fibrosis.

Model type	Approach	Species	Culture Conditions	Physical Parameters	Aim	Study
CL (multiwell) and OP (continuous flow in silicon tubes bioreactor)	Consortium	*P. aeruginosa S. aureus* (1:250)	Medium: BHI 20% (CL), BHI 10% (OP) Seeding: 7 × 10^7^ cells/ml (*S. aureus*) (OP and CL)	T: 37°C (CL), 22°C (OP) Replenishing rate (CL): 12 h Medium flow (OP): 10.8 ml/h Duration (*S. aureus*): 5 days Duration (coculture): 14 days Sampling Rate: 24 h	Dynamic competition	Woods et al., 2018
CL: multiwell, solid agar medium	Consortium	*P. aeruginosa S. aureus* (1:1)	Medium: (tailored) M14 Seeding: 10^8^ CFU/ml	T: 37°C Duration: 3 h (mono and cocultures)	Dynamic competition Genetic expression	Tognon et al., 2019
CL: multiwell, liquid medium	Consortium	*P. aeruginosa S. aureus* (1:100 and 1:1000)	Medium: (tailored) M14 Seeding: 5 (@#105 CFU per well (*P. aeruginosa*	T: 37°C Duration: 15 days Sampling rate: 24 h.	Adaptation to lung environment	Tognon et al., 2017
CL: solid agar medium	In between consortium and microcosm	*P. aeruginosa* (clinical isolate) *S. aureus* (lab strain) (1:1)	Medium: LB (*P. aeruginosa*), TSB (*S. aureus*) Seeding: [0.5;1] x 10^8^ CFU/ml	T: 37(°C Duration: 16 h	Genetic expression	Limoli et al., 2017
CL: multiwell, liquid	Consortium	*P. aeruginosa S. aureus*	Medium: LB (*P. aeruginosa*), TSB (*S. aureus*), MEM + L-Gln Seeding: 50 μl of 0.05 OD_600_ cell suspension (*S. aureus*), ans *P. aeruginosa* supernatant	T: 37°C CO_2_: 5% Duration: 30 h	Antibiotic resistance	Orazi and O’Toole, 2017
CL: bacteria cultured onto epithelial cell monolayers	Microcosm	*P. aeruginosa* (biofilm forming and non-forming strains) + epithelial cells	Medium: LB Seeding: 1.0 OD_600_ *P. aeruginosa* suspension diluted up to 1:500 and seeded on murine epithelial cells	T: 37°C Duration: 20 h	Biofilm formation	Woodworth et al., 2008; Cont et al., 2020

*CL, closed system; OP, open system; BHI, Brain Heart Infusion; M14, is a tailored medium ([137]); LB, Lysogeny Broth; TSB, Tryptic Soy Broth; MEM, Minimal Essential Medium.*

## Conclusive Remarks

The study of the biological features of polymicrobial infections needs to be considered to develop effective antimicrobial strategies useful to treat complex and chronic diseases like CF and bronchiectasis. The available *in vitro* testing methods provide the state-of-the-art although they were not developed to study the crosstalk among different bacteria and the effect of the three-dimensional environment. They represent important but limited examples available up-to-now: two-species cultures and biofilms as unique examples of polymicrobial cultures and 3D-environments, respectively. New developments in co-cultures and the study of *in vitro* bacterial three-dimensional substrates are needed to overcome the frontier for the production of realistic tools to be employed in the study of bacterial interactions and drug discovery and to switch to the reproduction of 3D environments and polymicrobial cultures. Within this scenario, we envision the need for *in vitro* methods that could impact on diverse applicative sectors.

## Author Contributions

MO and LZ worked on the draft of the document and revised the manuscript with the help of GG. LZ organized the bibliography. FB and SVU wrote the first draft of some sections. PP organized and focussed the contributions and coordinated the revision and the answers to the reviewers. PP and LV designed the content and the structure of the review and critically revised the manuscript in all stages. All authors contributed to the final manuscript revision, read and approved the submitted version.

## Conflict of Interest

The authors declare that the research was conducted in the absence of any commercial or financial relationships that could be construed as a potential conflict of interest.

## Abbreviations

BBC: Biofilm Bactericidal Concentration
BHI: Brain Heart Infusion
BPC: Biofilm Preventing Concentration
CF: cystic fibrosis
CFU: colony forming units
CL: closed system
CLSM: Confocal Laser Scanning Microscope
FISH: fluorescence *in situ* hybridization
LB: Lysogeny Broth
MBC: Minimal Bactericidal Concentration
MBEC: Minimal Biofilm Eradication Concentration
MBIC: Minimal Biofilm Inhibitory Concentration
MEM: Minimal Essential Medium
MIC: Minimal Inhibitory Concentration
MTT: 3-(4,5-dimethylthiazol-2-yl)-2,5-diphenyltetrazolium bromide
OP: open system
qPCR: Real Time-PCR
TSB: Tryptic Soy Broth
XTT: sodium 3 ′ -[1- (phenylaminocarbonyl)- 3,4- tetrazolium]-bis (4-methoxy6-nitro) benzene sulfonic acid hydrate.
